# Factor H autoantibodies and deletion of Complement Factor H-Related protein-1 in rheumatic diseases in comparison to atypical hemolytic uremic syndrome

**DOI:** 10.1186/ar4016

**Published:** 2012-08-15

**Authors:** Anna Foltyn Zadura, Peter F Zipfel, Maria I Bokarewa, Gunnar Sturfelt, Andreas Jönsen, Sara C Nilsson, Andreas Hillarp, Tore Saxne, Leendert A Trouw, Anna M Blom

**Affiliations:** 1 Department of Laboratory Medicine, Section of Medical Protein Chemistry, Lund University, Inga Marie Nilsson street 53, 205 02 Malmö, Sweden; 2 Department of Infection Biology, Leibniz Institute for Natural Product Research and Infection Biology, Beutenbergstrasse 11a, 07745 Jena, Germany; 3 Department of Rheumatology and Inflammation Research, University of Gothenburg, Guldhedsgatan 10A, 413 46 Gothenburg, Sweden; 4 Department of Clinical Sciences Lund, Section of Rheumatology, Lund University, Kioskgatan 3, 221 85 Lund, Sweden; 5 Department of Rheumatology, Leiden University Medical Center, PO Box 9600, 2300 RC Leiden, The Netherlands

## Abstract

**Introduction:**

Complement activation is involved in rheumatoid arthritis (RA), systemic lupus erythematosus (SLE) and atypical hemolytic uremic syndrome (aHUS). Autoantibodies to complement inhibitor factor H (FH), particularly in association with deletions of the gene coding for FH-related protein 1 (CFHR1), are associated with aHUS.

**Methods:**

Autoantibodies against FH, factor I (FI) and C4b-binding protein (C4BP) were measured by ELISA, while CFHR1 homozygous deletion was determined with Western blotting of sera. Epitopes for FH autoantibodies were mapped using recombinant fragments of FH.

**Results:**

FH autoantibodies were detected in SLE (6.7%, *n *= 60, RA patients (16.5%, *n *= 97 in the Swedish cohort and 9.2%, *n *= 217 in the Dutch cohort) and thrombosis patients positive for the lupus anticoagulants (LA+) test (9.4%, *n *= 64) compared with aHUS patients (11.7%, *n *= 103). In the control groups (*n *= 354), an average of 4% of individuals were positive for FH autoantibodies. The frequencies observed in both RA cohorts and LA+ patients were statistically significantly higher than in controls. We also found that an average of 15.2% of the FH-autoantibody positive individuals in all studied disease groups had homozygous deficiency of CFHR1 compared with 3.8% of the FH autoantibody negative patients. The levels of FH autoantibodies varied in individual patients over time. FH autoantibodies found in LA+, SLE and RA were directed against several epitopes across FH in contrast to those found in aHUS, which bound mainly to the C-terminus. Autoantibodies against FI and C4BP were detected in some patients and controls but they were not associated with any of the diseases analyzed in this study.

**Conclusions:**

Autoantibodies against FH are not specific for aHUS but are present at a significant frequency in rheumatic diseases where they could be involved in pathophysiological mechanisms.

## Introduction

Complement is a central innate defense system that promotes the inflammatory response and destroys microbes. In addition, complement is also involved in the instruction of the adaptive immune response and the clearance of dead cells and misfolded proteins [1,2]. Complement consists of plasma- and membrane-associated proteins and can be activated through the classical, the lectin and the alternative pathways [3].

Complement is an aggressive, self-amplifying cascade that needs to be tightly regulated by both soluble and membrane-bound inhibitors to prevent damage of host tissues. The soluble inhibitor C4b-binding protein (C4BP) has a central role in regulating the classical and the lectin pathways [4], while Factor H (FH) and its splice variant FH-like protein 1 (FHL-1) corresponding to complement control protein (CCP) domains 1-7 of FH are the most important soluble inhibitors of the alternative pathway [5]. Factor I (FI) is a serine protease that inhibits all complement pathways but works only in the presence of its specific cofactors, such as FH and C4BP [6,7]. Defective activation of complement as well as insufficient inhibition are associated with pathological processes in a number of autoimmune and inflammatory diseases [8] including rheumatoid arthritis (RA) [9], systemic lupus erythematosus (SLE) [10-12], anti-phospholipid syndrome (APS) [13] and atypical hemolytic uremic syndrome (aHUS) [14]. In addition to genetic variants, autoantibodies also have been reported to have an impact on the function of complement factors and on diseases [15]. It is now well established that the presence of autoantibodies against complement FH is associated with aHUS [16-20] and it was also reported that the deletion of complement FH-related proteins 1 and 3 (CFHR1/CFHR3) in aHUS patients are associated with the disease [21,22]. This autoimmune subtype of aHUS with unique characteristics was recently termed DEAP-HUS (the Deficiency of CFHR plasma proteins and Autoantibody Positive form of HUS) [23]. Interestingly most of the FH-autoantibodies in aHUS are directed against the C-terminal recognition region of FH [17].

In this study we have examined the frequency of FH-autoantibodies in groups of patients with different diseases, such as RA, SLE and thrombosis patients positive for lupus anticoagulants (LA+) test and compared these with an aHUS cohort. We have also investigated if the presence of those antibodies is associated with deficiency of CFHR1 and which regions of FH interact with autoantibodies.

## Materials and methods

### Patients and controls

Plasma samples from consecutive unselected patients with RA (*n *= 314) were collected in three centers: at the Department of Rheumatology, Lund University Hospital, Lund, Sweden (*n *= 30); the Department of Rheumatology and Inflammation Research, Gothenburg, Sweden (*n *= 67) and at the Department of Rheumatology, Leiden University Medical Center, Leiden, The Netherlands (*n *= 217). The RA samples from Sweden (Lund and Gothenburg) were analyzed as one cohort. All patients fulfilled the American College of Rheumatology criteria for RA [24]. Four of the FH-autoantibody positive patients from the Lund cohort were then chosen and the FH-autoantibodies were measured in several samples collected from these four patients, mainly after the first positive, analyzed sample.

Plasma samples from patients with SLE were collected at the Department of Rheumatology, Lund University Hospital, Sweden. From each patient (*n *= 30) two samples were available, selected from time points with lower (median = 12, range = 12) and higher disease activity (median = 32, range = 28) as measured by SLE disease activity index (SLEDAI). All SLE patients fulfilled four or more American College of Rheumatology classification criteria for SLE [25,26].

Plasma samples from thrombosis patients positive for lupus anticoagulants (LA; screen test) using dilute Russels Viper Venom [27] (dRVVT) (Siemens; Marburg, Germany) and thus with highly likely diagnosis of APS (*n *= 64) were collected at the Department of Clinical Chemistry, Skåne University Hospital Malmö, Sweden. Since the LA test was performed only on one occasion before the samples were de-identified, these patients do not fulfill Sydney criteria for APS diagnosis [28]. aHUS patients (*n *= 103) from the Jena registry [22] were recruited based on the initial clinical diagnosis aHUS.

Controls used were collected from unrelated healthy volunteers from Germany (*n *= 20) and The Netherlands (*n *= 161) and matched in average age and sex. The total number of 173 Swedish controls was divided into groups exactly matching in age and sex to each disease group (SLE, RA, LA+) for the analysis of FH autoantibodies. These controls were matched in average age and sex for the analysis of FI and C4BP autoantibodies. The study was approved by the regional ethics committees of Jena, Lund, Leiden and Gothenburg Universities and all participants have given informed consent.

### Determination of FH-, FI- and C4BP-autoantibodies levels and identification of autoantibody positive patients

Plasma samples from patients with RA, SLE, LA+, aHUS and controls were screened for the presence of FH-, FI- and C4BP-autoantibodies using enzyme-linked immunosorbent assay (ELISA). Microtiter plates (Maxisorp; Nunc, Roskilde, Denmark) were coated overnight with purified FH, FI and C4BP diluted to 1 μg/ml in 75 mM sodium-carbonate buffer, pH 9.6. FH was purified from plasma [29] and passed through protein A-Sepharose HiTRAP, (GE Healthcare, Uppsala, Sweden) to remove any contaminating human immunoglobulins. C4BP and FI were expressed recombinantly in human embryonic kidney 293 (HEK 293) cells and purified by affinity chromatography as described [30,31]. The plates were blocked with PBS/1% BSA for 1 h at 37°C after which plasma samples diluted 1:50 times in PBS/1% BSA/Tween 0.05% were added to the plates. After 1 h incubation at 37°C the plates were washed four times with washing buffer (50 mM Tris-HCl, 150 mM NaCl, 2 mM CaCl_2 _and 0.1% Tween 20; pH 8.0). FH-, FI- and C4BP-autoantibodies were detected with rabbit anti-human IgG Abs (DakoCytomation, Glostrup, Denmark) followed by swine anti-rabbit Abs conjugated with horseradish peroxidise (HRP) (DakoCytomation). The plates were developed with *o*-phenylenediamine (OPD) substrate (DakoCytomation) and the absorbance was measured at 490 nm (Varian 50 MPR Microplate Reader). Concentrations of FH-, FI- and C4BP-autoantibodies were calculated relative to a standard set at 100 AU/mL. Polyclonal goat antibodies against human FH (Quidel, San Diego, CA, USA) and rabbit antibodies against human FI (PK9205, generated in house) and human C4BP (PK9008, generated in house) were used as standards at two-fold dilution series starting from 1:8,000, 1:200 and 1:2,000, respectively.

**Figure 1:**
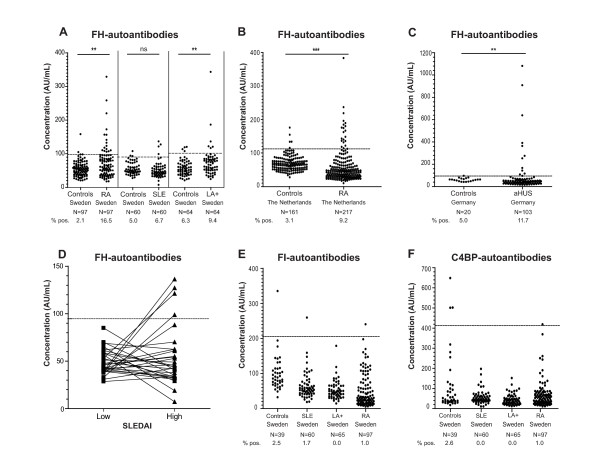
**Identification of FH, FI and C4BP autoantibody positive patients**. Plasma samples of LA+, SLE, RA, aHUS patients and healthy controls were analyzed for binding of IgG autoantibodies on immobilized purified FH (**A-D**), recombinant FI (**E**) or recombinant C4BP (**F**) using ELISA. FH-autoantibody titers for individual SLE patients characterized by presence of FH-autoantibodies divided according to SLEDAI score indicating low and high disease activity for the same patients are shown in Figure 1D. Values obtained for the same patient are connected by lines. The samples with levels above the mean plus 2 SD of those in the matched control group were considered positive. The dotted lines represent cut-offs calculated separately for each control group. Statistical significance of differences between autoantibody titers in diseases groups compared with controls was calculated using a Kruskal-Wallis test or Mann-Whitney test. *P*-values lower than 0.05 were considered statistically significant. ** *P *< 0.01; *** *P *< 0.001, ns not significant.

The samples with levels above the mean plus two standard deviations (SD) of those in the matched control group were considered positive and are indicated by dotted lines in each panel of Figure 1. The cut-offs were calculated separately for each country control group and for each control group matched to a Swedish disease group. The cut-offs for FH-autoantibodies were >98.7 AU/mL (RA, Sweden), >90.5 (SLE Sweden), >102.3 (LA+, Sweden), >113.2 AU/mL (RA, The Netherlands) and >89.2 AU/mL (aHUS, Germany). The cut-offs for FI-autoantibodies and C4BP-autoantibodies were >203.6 AU/mL and >407.2 AU/mL, respectively, based on the Swedish control group consisting of 39 healthy individuals.

To analyze the specificity of the assay, 8 μg/well of purified FH, FI, C4BP were added to several plasma samples with high autoantibody titers diluted 1:50. The samples were then pre-incubated at RT for 2 h before performing the analysis. We believe that the assay is not affected by the presence of rheumatoid factor because FH was directly immobilized on plates. In support, we found no association between positivity for rheumatoid factor and FH autoantibodies (not shown). An additional specificity control was performed by incubating polyvinylidene difluoride (PVDF) membrane fragment containing excised band corresponding to FH with serum of a patient positive for FH autoantibodies. The bound antibodies were eluted with 0.1 M glycin, 0.3 M NaCl pH 2.7, neutralized with Tris buffer and incubated with another PVDF membrane containing separated purified FH, pre-albumin and lysate of HEK 293 cells spiked with purified FH. Bound antibodies were then visualized by sequential incubation of the membrane with rabbit anti-human IgGs and goat anti-rabbit HRP conjugated antibodies, followed by development with a colorimetric substrate. The FH autoantibody assay has now been reproducibly established by several independent research groups [17,19,32].

### Determination of binding site for FH autoantibodies using FH fragments

Several serum samples positive for FH autoantibodies were chosen from each patient group to localize the binding domains. These were incubated with immobilized BSA, FH, FHL-1 and recombinant FH fragments expressed as described previously in a baculovirus system and composed of CCPs 8 to 20, CCPs 15 to 20 and CCPs 19 to 20 [17]. The fragments were immobilized at equivalent molar concentrations corresponding to 1 μg/ml FH and antibody binding was determined as described above.

### Western blot analysis

Plasma samples from 64 LA+, 60 SLE, 314 RA, 101 aHUS patients and 354 controls were investigated for the presence of CFHR1. Plasma samples diluted 1:100 were separated under non-reducing conditions using 12% SDS-PAGE. The proteins were transferred onto PVDF membrane, blocked with quenching solution (washing buffer: 50 mM Tris-HCl, 150 mM NaCl, 0.1% (v/v) Tween 20, pH 7.5 with 0.3% fish gelatin, Norland Products, Cranbury, NJ, USA) and incubated with mouse monoclonal anti-FH (C18/3; Santa Cruz Biotechnology, Santa Cruz, CA, USA) that identifies the conserved C-terminus of FH (150 kDa) and the two differentially glycosylated forms of CFHR1α and CFHR1β (37 and 42 kDa). Bound antibodies were detected with a polyclonal anti-mouse IgG antibody conjugated with HRP (DakoCytomation). Finally, the blots were developed using 3,3'-diaminobenzidine tetrahydrochloride (Sigma-Aldrich, St Louis, MO, USA) colorimetric substrate system as described before [33].

We have confirmed the capacity of the Western blot technique to reliably identify CFHR1 deletion by comparing 70 RA samples tested by both Western blot and genetic analysis using Mutiplex Ligation-dependent Probe Amplification (MLPA) (MRC-Holland, Amsterdam, the Netherlands) following the instructions from the manufacturer.

### Statistical analysis

Differences between the patients and controls were evaluated using Mann-Whitney test. *P*-values lower than 0.05 were considered statistically significant.

## Results

### FH autoantibodies are present in sera of patients with rheumatic diseases

Autoantibodies against FH (Figure 1A-D), FI (Figure 1E) and C4BP (Figure 1F) were analyzed in samples of LA+, SLE, RA and aHUS patients as well as in healthy controls using ELISA. The samples with levels above the mean plus 2 SD of those in the country as well as disease specific control groups were considered positive, as indicated by dotted lines in each panel of Figure 1.

In the Swedish control groups matched individually to RA, SLE and LA+ patients, only two (2.1%) healthy individuals were positive for FH-autoantibodies in the control group matched to RA patients, three (5%) in the group matched to SLE patients and four (6.3%) matched to LA+ patients. In the control groups of 161 (The Netherlands) and 20 (Germany) healthy individuals, 5 (3.1%) and 1 (5%) FH-autoantibody positive samples were detected. In the cohorts of LA+, SLE, RA and aHUS patients, we identified several patients positive for FH-autoantibodies (Figure 1A-D). The cohort of aHUS patients was used as a positive control and, indeed, we observed a statistically significant (*P *< 0.01) increase in frequency of FH-autoantibody positive individuals in aHUS as compared to the controls. The previously reported frequency of FH-autoantibodies in the German cohort of 147 aHUS patients was 11% (16/147) [17,18] compared with 9% (13/142) in the Newcastle cohort [19], which corresponds well with our results (11.7%) for aHUS patients.

Interestingly, also in the Swedish RA patients, a significantly increased (*P *< 0.01) frequency of FH-autoantibody positive patients was observed as compared to matched healthy controls (Figure 1A). Independent international replication was sought using a cohort of Dutch RA patients and their controls. Also, here an increased frequency of FH-autoantibody positive patients was observed (*P *< 0.001; Figure 1B). The frequencies observed were reaching up to 16.5% in the Swedish cohort and 9.2% in the Dutch cohort (Figure 1A and Table 1).

**Table 1:** Frequency of FH-autoantibodies and CFHR1 deficiency in patients compared to healthy controls.

Total number of patients and controls		FH-autoantibody positive individuals			FH-autoantibody negative individuals		Odds Ratio for anti-FH positivity in CFHR1 deficiency		
		%	**No**.	CFHR1 deficiency No. (%)	**No**.	CFHR1 deficiency No. (%)	OR	95% CI	*P*-value
LA+ Sweden	64	9.4	6	5 (83.3)	58	1 (1.7)	285	15.4 to 5,280	<0.0001
Controls Sweden	64	6.3	4	1 (25.0)	60	1 (1.7)	20	1.0 to 397	1.12
SLE Low SLEDAI, Sweden	30	0.0	0	0 (0.0)	30	1 (3.3)	n.a^1^		
Controls Sweden	30	0.0	0	0 (0.0)	30	0 (0.0)	n.a.		
SLE High SLEDAI, Sweden	30	13.3	4	0 (0.0)	26	1 (3.8)	2.0	0.066 to 54.1	1
Controls Sweden	30	10.0	3	0 (0.0)	27	1 (3.7)	6.3	0.17 to 231	1
RA Sweden	97	16.5	16	2 (12.5)	81	5 (6.2)	2.0	0.38 to12.3	0.3
Controls Sweden	97	2.1	2	0(0.0)	95	1(1.1)	12.3	0.44 to 384	1
RA The Netherlands	217	9.2	20	0 (0.0)	197	7 (3.6)	n.a.		
Controls The Netherlands	161	3.1	5	0 (0.0)	156	13 (8.3)	n.a.		
aHUS Germany	103	11.7	12	3 (25.0)	91	4 (4.4)	6.45	1.25 to 33.05	0.04
Controls Germany	20	5.0	1	0 (0.0)	19	0 (0.0)	n.a.		

^1^n.a., not applicable due to 0% frequency

Also in the group of LA+ patients, a positive signal for FH-autoantibodies was observed for 9.4% patients, which was higher compared to healthy controls (*P *< 0.01). Only 6.7% of the SLE plasma samples analyzed were positive for the presence of FH-autoantibodies, which was not significantly different from healthy controls (Figure 1A). FH-autoantibodies could only be detected in SLE-patients at the time point with higher disease activity (Figure 1D). The mean titer of FH-autoantibodies for all FH autoantibodies positive patients from the groups with rheumatic diseases was 161.2 AU/mL compared with 363.6 AU/mL for aHUS patients.

Furthermore, we did not observe a statistically significant correlation between FH-autoantibody titers and age (ranging from 16 to 94 years) or sex when analyzing all controls and patients together or separately.

In order to test if the studied diseases are characterized by autoantibodies against several complement inhibitors or only against FH, we also analyzed autoantibody reactivity against complement inhibitors C4BP and FI. The results from the ELISA measurement of FI- and C4BP-autoantibodies in cohorts of LA+, SLE, RA (Swedish cohort) showed that there is no obvious association between these autoantibodies and any of the rheumatic conditions investigated (Figure 1E, F). Likewise, we did not find an association between FI- and C4BP-autoantibodies and aHUS (not shown).

### FH autoantibody status varies during the course of disease

Four FH-autoantibody positive RA patients, from the Lund cohort, were analyzed for variation of FH-autoantibodies positivity varies during the disease course. The disease duration for the chosen patients varied between 0.5 to 35 years and also the time interval between the first and last collected sample ranged from 3 months to 22 years. Two patients had no detectable FH-autoantibodies in all samples drawn after the initial, FH-autoantibody positive sample. For the third patient, the first FH-autoantibody negative sample was followed by a positive one and then seven negative ones, whereas the last patient had FH-autoantibodies in all three samples tested. Together with the observed difference for SLE patients with high and low SLEDAI index, these results show that the FH-autoantibody status is not constant during the disease's progress.

### Determination of binding site for FH-autoantibodies using FH fragments

**Figure 2:**
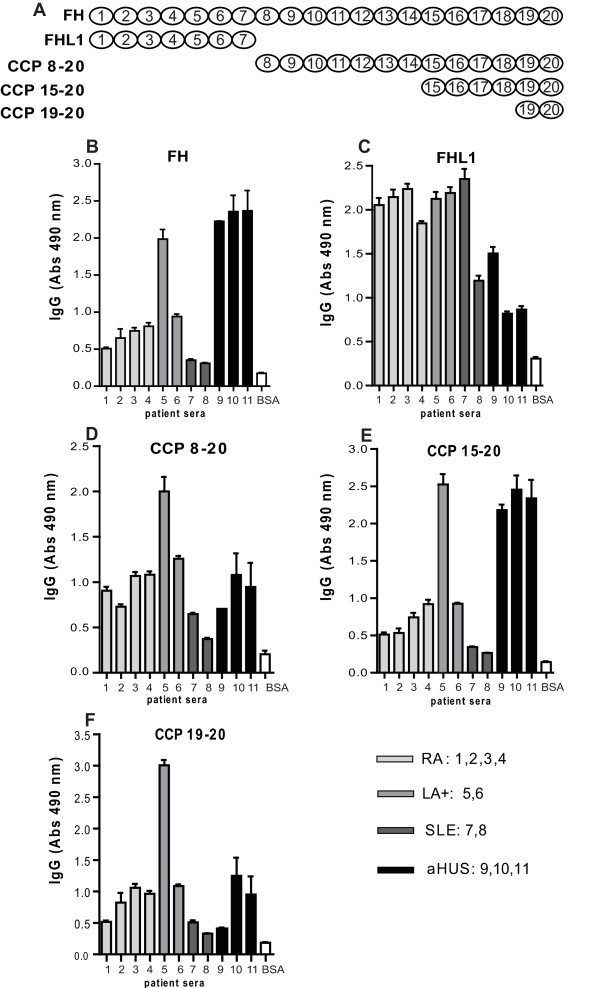
**Binding of autoantibodies to FH fragments**. Molar equivalent concentrations of the FH fragments (corresponding to CCPs 8 to 20, CCPs 15 to 20, CCPs 19 to 20), FHL-1 (CCPs 1 to 7), FH and BSA (negative control) were coated onto ELISA plates and autoantibody binding was determined with rabbit anti-human IgG antibodies followed by swine anti-rabbit HRP antibodies. The FH fragments used are schematically presented in panel **A**. Results are shown as mean values of three independent experiments +/- SD.

In order to determine the regions of FH that are recognized by autoantibodies, we analyzed by ELISA a few positive samples from each disease group and several fragments of FH and FHL-1. We confirmed that all selected samples contained autoantibodies reacting with intact FH with the highest levels found in the three selected aHUS patients and one LA+ patient (Figure 2B). We found that FH-autoantibodies present in aHUS/DEAP-HUS patients bound mainly to the C-terminus of FH as previously reported [17], since we detected the strongest signals of the three serum samples of aHUS patients for intact FH, CCPs 15 to 20 and CCPs 19 to 20 but very low level of interaction with FHL-1, which contains only CCP1 to 7 (Figure 2). To the contrary, samples from RA, LA+ and SLE reacted in a similar manner with all used fragments of FH, including FHL-1. This indicated that FH-autoantibodies found in rheumatic diseases lack specificity for the C-terminus of FH as found in DEAP-HUS.

### CFHR1 deficiency in patients with rheumatic diseases and aHUS

**Figure 3:**
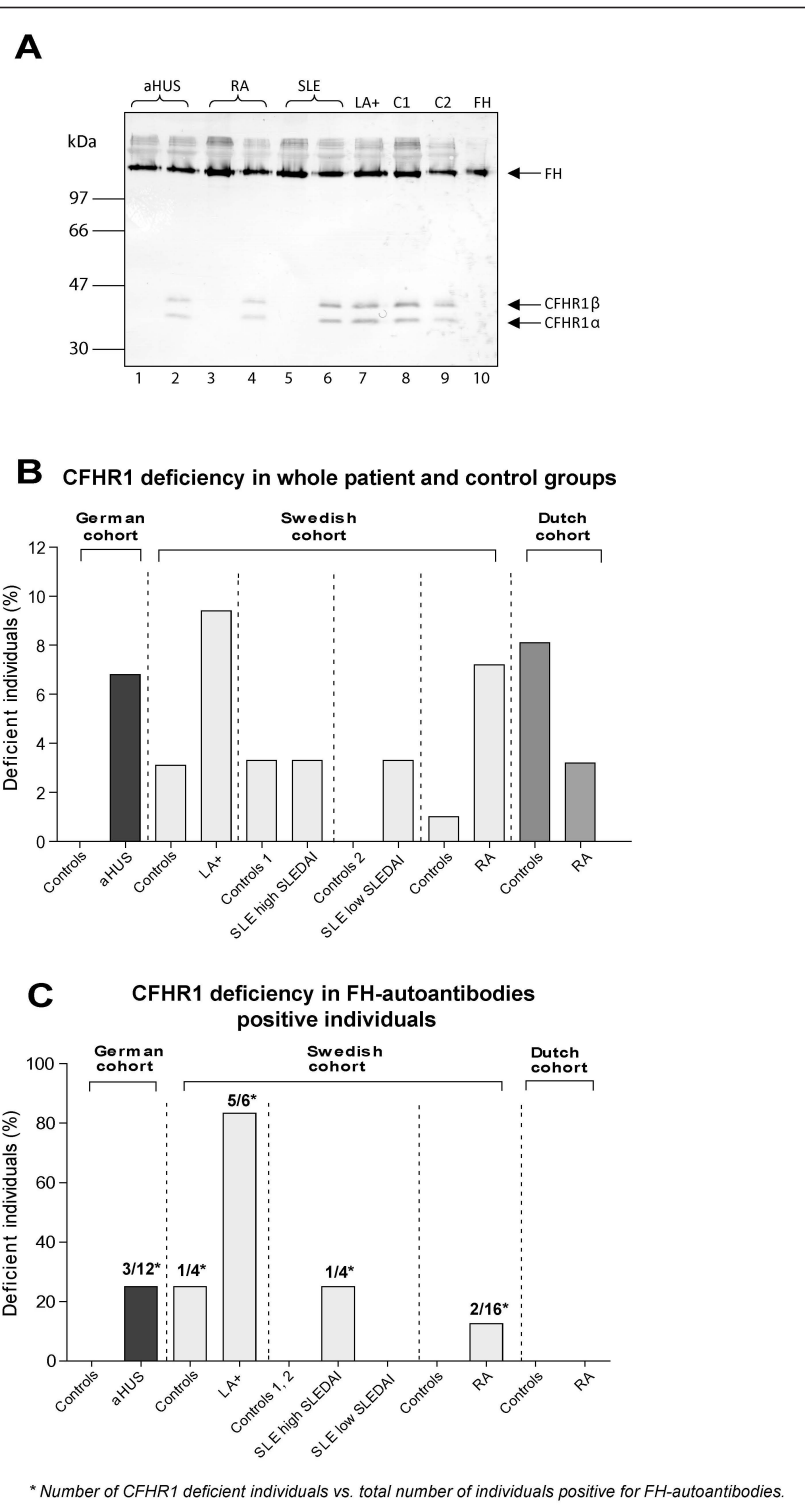
**Complete homozygous deficiency of CFHR1**. **A**) Plasma samples of patients and healthy controls were separated on 12% SDS-PAGE, transferred to a PVDF membrane, and analyzed by Western blotting using a mouse monoclonal antibody against FH (C18/3) that identifies FH (150 kDa) and the two differently glycosylated forms of CFHR1α and CFHR1β (37 and 42 kDa). Plasma samples of healthy controls are shown in lanes 8 and 9. CFHR1 positive patients were found for aHUS (lane 2), RA (lane 4), SLE (lane 6) and LA+ (lane 7). Patients with total CFHR1 deficiency are shown for aHUS (lane 1), RA (lane 3) and SLE (lane 5). Lane 10 shows purified FH. **B**) Complete CFHR1 deficiency in whole patient groups (including FH-autoantibody positive and negative patients) determined using Western blotting. **C**) Complete CFHR1 deficiency in FH-autoantibody positive individuals in different disease and control groups. Numbers indicated above bars in the panel C represent number of CFHR1 deficient patients vs. total number of patients positive for FH-autoantibodies.

Since in aHUS an association was reported between the presence of FH-autoantibodies and homozygous deletions of the gene encoding CFHR1 and CFHR3 [22], we now also analyzed whether there is such an association in rheumatic diseases. Previously, a good correlation was shown between the genetic deficiency of CFHR1 and the lack of CFHR1 protein detected by Western blotting in samples of aHUS patients [34] and thus we used the latter analysis to determine the frequency of CFHR1 deficiency. We confirmed the CFHR1 deficiency on Western blot with CFHR1 genetic deficiency in 70 RA patients using MLPA and observed a 100% match between the two techniques. The Western blot shows both FH (approximately 150 kDa) and the two glycosylated forms of CFHR1α and CFHR1β (approximately 38 and 43 kDa). A typical Western blot analysis showing examples of CFHR1 positive and deficient patients is shown together with controls (Figure 3A). A complete homozygous CFHR1 deficiency was found in 5.4% of healthy controls, which is in agreement with previous reports [18,22].

A frequency of CFHR1 deficiency, when analyzing all patient groups used in this study (CFHR1 deficient persons in FH-autoantibody negative and positive groups), was comparable with that in the aHUS cohort (Figure 3B). However, when analyzing the FH-autoantibody positive patients separately we observed that 12.5 to 83.3% of patients with LA+, SLE, RA and aHUS characterized by FH-autoantibodies entirely lacked CFHR1 (Table 1, Figure 3C). The Dutch RA patients were an interesting exception with 0% CFHR1 deficiency in the FH-autoantibody positive patients.

It was shown previously that 77 to 88% of aHUS patients with FH-autoantibodies were deficient for CFHR1 and CFHR3 proteins [22]. In this study, we found that 15.2% (7/46) of the patients with LA+, SLE and RA positive for FH-autoantibodies had homozygous deficiency of CFHR1 compared with 25.0% (3/12) of FH-autoantibodies positive aHUS patients. There was no difference between frequencies of the deficiency of CFHR1 in patients with rheumatic diseases negative for FH-autoantibodies (3.8%; 15/392) compared with FH-autoantibody negative aHUS patients (4.4%; 4/91) (Table 1). We found that there was a powerful association between FH-autoantibody positivity and CFHR1 deficiency in LA+. Odds ratio measured as an increased risk for FH-autoantibody positivity depended on CFHR1 deficiency, estimated using Fisher's exact test was OR = 285 (95% CI 15.4 to 5,280, *P *< 0.0001) for LA+ and OR = 6.5 (95% CI 1.25 to 33.05, *P *= 0.04) for aHUS cohort (Table 1). Thus, it appears that generation of FH-autoantibodies is specifically associated with deficiency of CFHR1 irrespectively of which disease group is analyzed.

## Discussion

A pathologic association between FH autoantibodies and DEAP-HUS has been reported in several cohorts but the prevalence of these antibodies has not been assessed in other diseases. Due to involvement of complement in SLE, RA and LA+/APS and a frequent presence of various autoantibodies in these diseases, we have performed a pilot study evaluating frequency of FH autoantibodies in these patient groups. Furthermore, we assessed prevalence of autoantibodies directed against two other soluble complement inhibitors C4BP and FI. We found no association of C4BP or FI autoantibodies and RA, SLE or LA+. To the contrary, significant increase in frequency of FH-autoantibodies compared to matched controls was found not only as previously reported in aHUS but also in RA and LA+.

The analytical specificity for these three autoantibody assays was evaluated by inhibition experiment using excess antigen. Addition of excess purified FH, FI or C4BP to several samples with high autoantibody titers diminished the signal (not shown) indicating specificity of the autoantibodies and demonstrating that the binding of these autoantibodies is not restricted to plastic absorbed protein. Furthermore, RA patient antibodies eluted from a PVDF membrane fragment containing FH transferred from SDS/PAGE gel, recognized purified FH but not pre-albumin separated by SDS/PAGE and transferred to a PVDF membrane (not shown). Incubation of HEK293 cell lysate spiked with FH with such specifically eluted patient FH-autoantibodies revealed mainly the signal corresponding to FH (150 kDa) and only very minor signals for bands around 270 kDa and 50 to 60 kDa. Thus, we concluded that FH-autoantibodies appear to be specific for FH and do not react with other human proteins.

FH is the main soluble inhibitor of the alternative complement pathway due to several mechanisms [5]. FH acts as cofactor to a serine protease FI in a degradation of C3b but it also inhibits the formation and accelerates the decay of the alternative pathway C3-convertase. The main region responsible for these activities is N-terminal fragment composed of CCP1-4. Furthermore, C-terminal CCPs 19 to 20 are crucial for the attachment of FH to cellular surfaces in order to provide protection. The mechanism by which FH autoantibodies contribute to aHUS is still under investigation but several interesting observations have been made. In most cases reported so far, FH autoantibodies were directed against CCP19 to 20 of FH and did not inhibit complement inhibitory activity of FH in the fluid phase but rather blocked binding of FH to C3bBb convertase [16] and to cell surfaces [17]. The C-terminus of FH is also the region in which most of the aHUS-associated mutations are located. It has been suggested that under conditions of enhanced complement activation, a lower local concentration of FH at the cell surface may lead to cell damage. In case of aHUS, the main targeted tissue may be endothelium. In rheumatoid arthritis, joint inflammation causes exudation of plasma proteins including complement factors and inhibitors into synovial fluid. There is strong evidence of ongoing activation of complement in synovial fluid [9], stimulated among others by molecules released during inflammation from the cartilage, such as fibromodulin [35] and cartilage-oligomeric matrix protein [36]. FH has been shown to attenuate complement activation initiated by these molecules [37] and autoantibodies against FH, which we now observed in RA patients, also bind to and functionally impair the N-terminus of the protein, which may result in enhanced complement activation, ensuing inflammation and tissue damage in joints.

We found that RA patients from two independent cohorts have significantly increased FH autoantibody frequency compared to controls. We did not find any positive correlation between FH-autoantibody positivity and type of treatment and other analytical data in the Swedish RA cohort. Analysis of the Dutch RA patients did not reveal any significant differences between the FH-autoantibodies positive- and negative patients, regarding sex, age, autoantibody status, inflammation or smoking. However, the low number of FH-autoantibody-positive individuals highly limits the power to reliably find such differences. Furthermore, the presence of FH-autoantibodies in several chosen RA patients varied during disease course but it was not associated with infections. A larger study is required to find out if FH-autoantibodies are associated with disease flares or severity of RA.

When analyzing samples from SLE patients, FH-autoantibodies could only be detected at the time point with higher disease activity but no significant correlation between FH-autoantibody titers and disease activity (SLEDAI), or with any particular SLE ACR criteria were found. This may be due to the low power to detect differences with only a few FH-autoantibody positive patients and a low overall number of patients in this exploratory study. Interestingly, in DEAP-HUS FH autoantibodies were clearly lower at remission than at disease onset [32]. Potential association of FH autoantibodies with nephritis would be of particular interest to study further due to recently reported genetic association with FH polymorphisms and SLE [38], as well as association with non-synonymous mutations in FH and CD46 with faster onset of nephritis in SLE patients [39].

So far little information on prevalence of mutations or polymorphisms in complement inhibitors, such as FH, has been published for RA or APS. A recent study found no association between common FH polymorphisms predisposing to age-related macular degeneration and RA [40]. However, our current data together with previously published observations suggest that generation of FH-autoantibodies is specifically associated with deficiency of CFHR1 in many different disease groups. So far there is no clear explanation for the association between these two phenotypes. It has been suggested that autoantibodies generated in the context of a CFHR1 deficiency are targeted to a region of the FH molecule that is critical for the development of aHUS, that is, CCP19 to 20, and somehow is related to structural and functional similarity between CFHR1 and FH [41]. However, this is not the case for the RA patients in the current study who carry FH-autoantibodies against many different regions of FH. To what extent this is the effect of intra-molecular epitope spreading or *de-novo *recognition of epitopes outside CCP19 to 20 remains unknown. However, our results suggest that FH-autoantibodies found in RA, SLE and LA+ patients are polyclonal in nature since they interact with several different regions of FH. Another conclusion from these data could be that the combination of a deletion of CFHR1 and autoantibodies against FH is a risk factor for APS, or at least positivity for the lupus anticoagulants' test. The number of patients analyzed in this study was limited to support such strong conclusions and future replication studies in well-defined thrombosis and APS patients will have to be performed to support this observation.

## Conclusions

Our study is the first to show that FH-autoantibodies can be observed in disorders other than aHUS, such as in patients suffering from rheumatic diseases. It has been shown that, in DEAP-HUS, FH-autoantibodies block the C-terminal recognition domain of FH [17], which results in less binding of FH to cell surfaces leading to reduced complement inhibition at these surfaces. This process is thought to play an important role in the glomeruli of patients suffering from DEAP-HUS during the episode of active disease and it is plausible that a similar process will also impact on tissue damage during episodes of active disease in patients suffering from these rheumatic conditions. The epitope mapping experiments suggest that the FH-autoantibodies observed in rheumatic diseases may bind to several epitopes scattered over FH. Antibodies against the N-terminus of FH may impair its ability to inhibit complement both on surfaces and in body fluids leading to a more generalized inflammation observed in rheumatic conditions in comparison to aHUS.

## Abbreviations

aHUS: atypical hemolytic uremic syndrome; APS: anti-phospholipid syndrome; C4BP: C4b-binding protein; CCP: complement control protein; CFHR1/CFHR3: complement FH-related proteins 1 and 3; DEAP-HUS: the Deficiency of CFHR plasma proteins and Autoantibody Positive form of HUS; dRVVT: dilute Russels Viper Venom; ELISA: ELISA, enzyme-linked immunosorbent assay; FH: factor H; FI: factor I; HRP: horseradish peroxidase; LA: lupus anticoagulants; MLPA: Mutiplex Ligation-dependent Probe Amplification; OPD: *o*-phenylenediamine; PVDF: polyvinylidene difluoride; RA: rheumatoid arthritis; SLE: systemic lupus erythematosus; SLEDAI: SLE Disease Activity Index.

## Competing interests

The authors declare that they have no competing interests.

## Authors' contributions

AFZ and SCN performed the research. AFZ, MB, GS, SCN, TS, LAT and AMB analyzed the results. PFZ, MB, GS, AJ, AH, TS and LAT provided patient data. PFZ contributed vital reagents. AFZ, AMB and LAT designed the study and wrote the paper. All authors read and approved the final manuscript.
